# CiliateGEM: an open-project and a tool for predictions of ciliate metabolic variations and experimental condition design

**DOI:** 10.1186/s12859-018-2422-9

**Published:** 2018-11-30

**Authors:** Alessio Mancini, Filmon Eyassu, Maxwell Conway, Annalisa Occhipinti, Pietro Liò, Claudio Angione, Sandra Pucciarelli

**Affiliations:** 1 School of Biosciences and Veterinary Medicine, University of Camerino, Camerino, Italy; 2 Department of Computer Science and Information Systems, Teesside University, Middlesbrough, UK; 3 Computer Laboratory, University of Cambridge, Cambridge, UK

**Keywords:** Ciliates, *Tetrahymena thermophila*, Genome scale reconstruction, Flux balance analysis, Metabolic pathways

## Abstract

**Background:**

The study of cell metabolism is becoming central in several fields such as biotechnology, evolution/adaptation and human disease investigations. Here we present CiliateGEM, the first metabolic network reconstruction draft of the freshwater ciliate *Tetrahymena thermophila.* We also provide the tools and resources to simulate different growth conditions and to predict metabolic variations. CiliateGEM can be extended to other ciliates in order to set up a meta-model, i.e. a metabolic network reconstruction valid for all ciliates.

Ciliates are complex unicellular eukaryotes of presumably monophyletic origin, with a phylogenetic position that is equal from plants and animals. These cells represent a new concept of unicellular system with a high degree of species, population biodiversity and cell complexity. Ciliates perform in a single cell all the functions of a pluricellular organism, including locomotion, feeding, digestion, and sexual processes.

**Results:**

After generating the model, we performed an in-silico simulation with the presence and absence of glucose. The lack of this nutrient caused a 32.1% reduction rate in biomass synthesis. Despite the glucose starvation, the growth did not stop due to the use of alternative carbon sources such as amino acids.

**Conclusions:**

The future models obtained from CiliateGEM may represent a new approach to describe the metabolism of ciliates. This tool will be a useful resource for the ciliate research community in order to extend these species as model organisms in different research fields. An improved understanding of ciliate metabolism could be relevant to elucidate the basis of biological phenomena like genotype-phenotype relationships, population genetics, and cilia-related disease mechanisms.

**Electronic supplementary material:**

The online version of this article (10.1186/s12859-018-2422-9) contains supplementary material, which is available to authorized users.

## Background

A metabolic network represents in an organism the complete set of biochemical reactions suitable to synthesise or break-down metabolites. These reactions drive the production of biomass and energy to support all cellular processes. The reconstruction of a full metabolic network occurring within each cell has advanced from early biochemical studies to algorithmically-generated pathway diagrams starting from genomic sequencing [1]. Genome-scale models (GEMs) offer a comprehensive exploration and a rapid analysis of genomic data. GEMs have been used extensively to study metabolic engineering [2, 3], model-driven drug discoveries [4, 5], prediction of cellular phenotypes after perturbations [6, 7], analysis of evolutionary processes [8–11] and models of interspecies interactions [12]. Organism-specific reconstructed metabolic networks may be further implemented to build mathematical models capable of simulating metabolic fluxes [13]. GEM pathway reconstruction has been used in *Caenorhabditis elegans* to predict genes essentiality [14] and to better understand the biology of arthropods [15], including those with a negative impact (vectors of human or animal diseases, agricultural pests). The latter approach is particularly useful to control harmful species and to develop new precautionary strategies [15]. Genome-scale metabolic modelling has also been successfully applied to study metabolic networks in microbes [16], including a Polychlorinated Biphenyl-degrading *Pseudomonas* [17, 18], thermophilic bacteria [19] and members of the human gut microbiota [20].

Among eukaryotic microbes, at least 25 models of *Saccharomyces cerevisiae* have been published since 2003 [21], helping to understand yeast metabolism. Ciliated protozoans may represent an alternative and useful eukaryotic model. Ciliates have been the main subject of projects supported by the EU Framework Programme Horizon 2020 such as the COST Actions. Moreover, the National Centre for Genome Resources and the Gordon and Betty Moore Foundation’s supported the Marine Microbiology Initiative (MMI). MMI created a valuable benchmark against the analysis of environmental transcriptomic data [22]. Ciliates, as yeasts, are eukaryotic single cells, but their structural and functional complexity is comparable to human and other metazoan cells. These unicellular organisms are similar to differentiated animal cells with complex functions and membrane-bound structures [23]. The potential number of extant ciliate species has been estimated at nearly 30,000 [24]. They represent an important mediator in the food chain by transforming ultrafine organic matter useful for zooplankton. Being individual cells, they are directly exposed to environmental changes, making them good models for studying cell-stress response and adaptation. Ciliates propagate mainly asexually by transverse fission, even though they perform conjugation, a sexual process that “renews” the genetic material. The complexity of ciliates is further represented by the presence of two different nuclei: the diploid micronucleus involved in conjugation and the polyploid macronucleus. While the first represents the germinal line, the second represents the somatic line. The macronucleus is responsible for gene expression during the vegetative phase.

We describe below a preliminary open software tool (*CiliateGEM*), focused on the *T. thermophila* macronuclear genomic sequences, which allows ciliates researchers to analyse a reconstructed network via Flux Balance Analysis (FBA). By studying this ciliate, we could discover new mechanisms for evolution and adaptation within metabolism, population, species and host-symbiont association. Cellular responses described in *T. thermophila* can be of fundamental importance to understand the biology of all ciliates. *CiliateGEM* is to date the most advanced tool available for ciliates and contains the highest number of curated biochemical reactions.

## Results

### CiliateGEM: An open project and a methodological pipeline

The complexity of ciliates makes it necessary to adopt a new approach to study their metabolism. To understand these organisms is fundamental to consider that they can feed, move, and reproduce in a single cell. Ciliate complexity includes also cell compartmentalisation, which ensures the optimal environment for each specific metabolic reaction (i.e. optimal lysosomes pH for macromolecule hydrolysis).

**Fig. 1:**
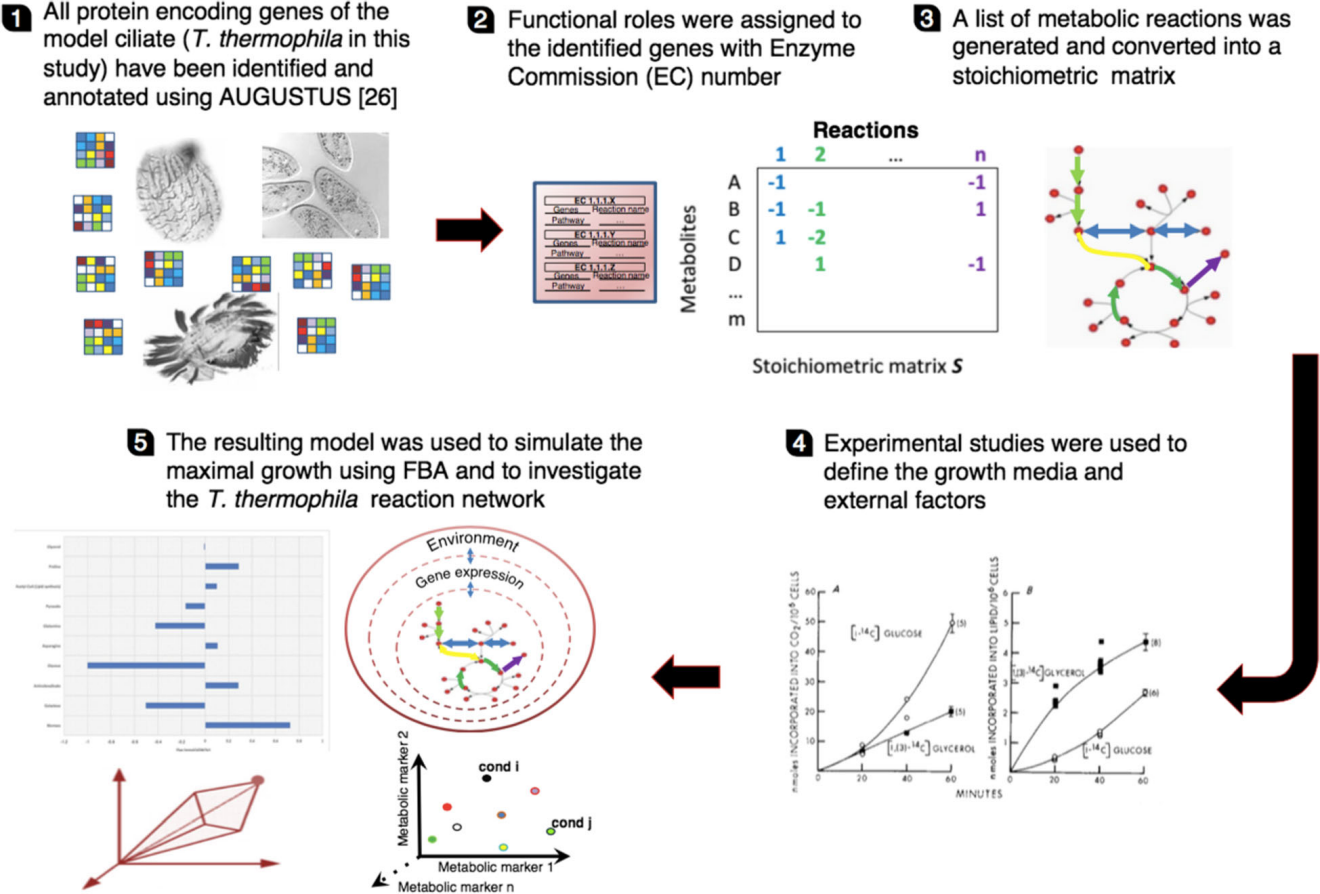
CiliateGEM pipeline. *CiliateGEM* was constructed using the protocol by Thiele and Palsson [13], as well as manual curation. To characterise the metabolic networks, we have gathered all core pathways from different organisms, including bacteria. *CiliateGEM* is provided in SBML and Matlab format as Additional files 6 and 7

To date, *CiliateGEM* allows the analysis of their reconstructed networks using the ciliate *Tetrahymena thermophila* as a case study*.* We choose this organism because *Tetrahymena* micro [25] and macro-nuclear [26] genomes have been sequenced and its biology has been extensively studied. The goal of this open project is to create a tool to simulate the growth of *Tetrahymena* cells in different conditions and then to expand it to all ciliates. The development and the refinement of this tool, coupled with the COBRA toolbox [27], can lead researchers to predict gene essentiality and genotype-phenotype relationship. *CiliateGEM* has been obtained using a bottom-up approach, from genome annotation to a mathematical model. The steps we followed are represented in Fig. 1.

### Tetrahymena growth simulation

One of the first experimental in vitro studies of metabolic pathways in ciliates were performed using *Tetrahymena pyriformis* as a model organism [28–30]*. Tetrahymena* cells were grown in a standard mixture of ^l4^C radiolabelled substrates including glucose, glycerol, pyruvate and glutamate. The incorporation of ^l4^C in CO2, glycogen and nucleic acids was measured with ranges of values depending on the carbon labelled position in the substrate structure. Borowitz et al. [29] reported a higher incorporation of ^l4^C into CO2 after 1 h of incubation with [^l4^C]glucose than in the same experiment with [^l4^C]glycerol, i.e. 43.9–64.2 nmol/10^6^ cells versus 19.8–19.9 nmol/10^6^ cells. Stein et al. [30] further reported a higher radiolabelled C incorporation in CO2 from [^l4^C]glucose than from pyruvate, glutamate and glycerol. The measured values of the labelled carbon incorporation into the CO_2_ product were as follows: [^l4^C]glucose (106–255 nmol/10^6^ cells); [^l4^C]pyruvate (55.4–121 nmol/10^6^ cells); [^l4^C]glutamate (1.90–3.96 nmol/10^6^ cells); [^l4^C]glycerol (11.0–13.6 nmol/10^6^ cells). Then, they tested glycogen as product, and the [^l4^C]glucose resulted the preferred substrate with the highest incorporation rates (313–496 nmol/10^6^ cells). A comparable incorporation rate between glycerol and pyruvate was reported (~ 6.5 nmol/10^6^ cells), as well as an absent incorporation from glutamate (< 0.2 nmol/10^6^ cells). The results differed from those of CO2 among the secondary substrates (glutamate, pyruvate and glycerol), but agreed on the highest used substrate (glucose).

**Fig. 2:**
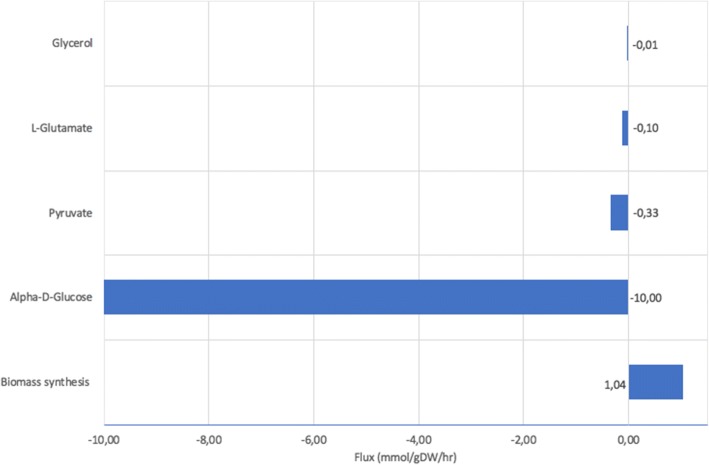
Rate of biomass synthesis by CiliateGEM from different substrates. The *CiliateGEM* model was allowed to utilise different carbon sources for growth. Glucose, glutamate and pyruvate consumption (illustrated by negative flux) directly affects the growth rate of *CiliateGEM* (depicted by positive flux values)

**Fig. 3:**
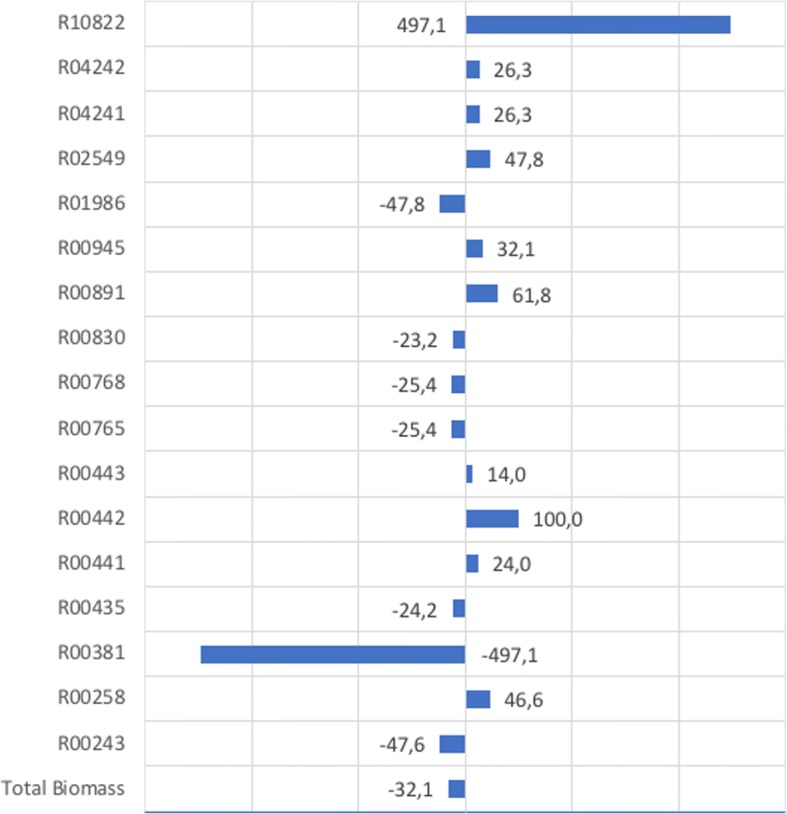
Differential biomass production (%) after glucose starvation. Values for growth with (Gg) and without glucose (Gwg) were used in this formula (Gwg-Gg)/|Gg|*100

Our initial simulation showed the maximum growth when glucose, glutamate, glycerol and pyruvate were used as growth media. In agreement with literature [29, 30], simulations of CiliateGEM showed that glucose is utilised as the preferred carbon source (Fig. 2). The uptake for glucose in our model (− 10 mmol/gDW/hr) is proportionally much higher than those of the secondary substrates (glutamate, pyruvate and glycerol) described by the authors in the previous in-vitro experiments [29, 30]. We allowed such uptake to saturate the system so the full amount is not necessarily utilised because of the thermodynamics constraints. Glucose is metabolised through glycolysis in the cytosol. A simulation in the presence of the above metabolites but without glucose showed a 32.1% reduction rate on biomass synthesis (Fig. 3). Despite the reduced biomass production, the growth without glucose is largely maintained by utilising alternative carbon sources such as amino acids (Fig. 3), as shown by the higher increase of biomass production for reactions R00891 (i.e. L-Serine + Hydrogen sulfide <= > L-Cysteine + H2O) and R00258 (L-Alanine + 2-Oxoglutarate <= > Pyruvate + L-Glutamate).

After glucose starvation, reactions R10822 [ADP + DNA(n) + 5’-Phospho-DNA(m) < => AMP + Orthophosphate + DNA(n + m)] and R00381 [ATP + DNA(n) + 5’-Phospho-DNA(m) < => AMP + Diphosphate + DNA(n + m)] showed the highest positive and negative values. Both reactions are catalysed by DNA ligases during DNA repair or recombination. These two reactions differ only on ATP or ADP cofactors. ATP is the phosphorylated form of ADP and is the preferred cofactor because phosphoanhydride bonds store a high amount of energy. Since ATP is synthesised from ADP during glycolysis by phosphorylation, we can speculate that in absence of glucose DNA ligation by ligase is performed by using ADP molecules as an alternative. DNA ligase using ADP has been reported from the aerobic hyper-thermophilic archaeon *Aeropyrum pernix* K1 [31].

Other opposite values were reported for reactions R02549 (4-Aminobutyraldehyde + NAD+ + H2O < => 4-Aminobutanoate + NADH + H+) and R01986 (4-Aminobutyraldehyde + NADP+ + H2O < => 4-Aminobutanoate + NADPH + H+), both involving in the arginine-catabolism pathway. Like R10822 and R00381 they only differ on cofactors, in this case NAD and NADP. While the structural difference between these two molecules is only the phosphate group, NADH participates in catabolic reactions, i.e. reactions that break down molecules to release energy, while NADPH participates in anabolic reactions, namely those that consume energy in order to build up or synthesise larger molecules.

Finally, reactions R04241 (ATP + THF-polyglutamate(n) + L-Glutamate <= > ADP + Orthophosphate + THF-polyglutamate(n + 1)) and R04242 (THF-polyglutamate + n H2O < => Tetrahydrofolate + n L-Glutamate) showed an increase of biomass production in the simulation without glucose. Both reactions are involved in folate biosynthesis, an essential cofactor for DNA and amino acid synthesis.

## Discussion

Studies on *Tetrahymena* have led ciliate researchers to landmark discoveries on cellular mechanisms, including the first cytoskeletal motor [32] and programmed translational frameshifting [33]. Some other studies have been awarded two Nobel prizes: to T. Cech and S. Altman for the discovery of catalytic RNA in 1989; and to E. H. Blackburn, C. W. Greider and J. W. Szostak for their work on telomeres in 2009. In general, mechanisms first described in ciliates have proven to be of widespread occurrence and of fundamental importance for the biology of all eukaryotes. Furthermore, ciliates are the only unicellular organisms in which programmed DNA elimination during somatic differentiation, widespread in animals [34], has been deeply characterised [35].

In this study, we set the basis of the open project *CiliateGEM* and we constructed a metabolic model draft of *T. thermophila*, made freely available to researchers. We checked the model robustness by simulating the organism cell growth in the presence or absence of glucose (the preferred carbon source utilised by this organism). Then we compared glucose to glutamate, pyruvate and glycerol consumption. Despite the organisms shows a decreased rate of biomass production, the growth without glucose is maintained by using alternative carbon sources, as amino acids and the tetrahydrofolate synthesis (essential cofactor in several metabolic reactions). This metabolic variation appears to increase the activity of ADP dependent DNA ligase I, possibly involved in DNA repair or recombination (R10822). We also speculated that in absence of glucose, ADP may be the preferred energy molecule instead of ATP, and the metabolism is switched towards catabolic reactions in order to obtain energy for growth. Our results showed consistency with previous studies on *Tetrahymena pyriformis* [28–30], therefore suggesting that CiliateGEM could provide a framework for the entire community.

*CiliateGEM* is freely available for academics and can be fully customised and updated. Our long-term objective is to achieve a “meta-model” that can be applied for metabolic simulations using other ciliates genomic resources (Additional file 1). The impact of *CiliateGEM* is to allow researchers to predict the response to changes in experimental conditions (e.g. starvation), and consequently to optimise the design of experimental protocols. Although this study focuses on *T. thermophila*, the findings may be shared with other ciliates as *Paramecium* and *Euplotes* species, including Antarctic strains [36]. In the future, we aim to reconstruct a multi-compartment model in symbiosis with algae by incorporating our datasets with an algal genome-scale model [37] (53% of reactions in the *CiliateGEM* are common in algae). Moreover, the complex systems of ciliates membranous compartments associated to phagocytosis and trafficking is strictly correlated with nutrients uptake and metabolism [38, 39]. We believe that understanding the role of compartments is even more important in multi compartmentalised unicellular organisms than in multicellular organisms (a list of ciliate organelles is reported in Additional file 2: Table S2). Reactions from different ciliates can be integrated in order to build up a hypothesis-driven scalable metabolic model based on common reactions.

## Conclusions

In conclusion, our work confirms that *Tetrahymena* can be used as model organism also for metabolic network reconstruction. Even though *Tetrahymena* is much less known from the metabolic point of view than others model organisms such as *Saccharomyces* and *C. elegans* (including the metabolic network operating during grown at laboratory-controlled conditions), the availability of “omic” resources allows reliable modelling of metabolic fluxes. Tools and resources obtained for *Tetrahymena* can then be applied to other ciliates and can elucidate genotype-phenotype mapping. A key point of this project is to create a collaborative network useful to expand and validate CiliateGEM with in vitro experiments on metabolic reactions already collected inside the model, and ultimately add new reactions. Collaboration between research teams in this type of projects is essential to speed up the process and to join the expertise of different research groups. A consensus modelling approach, already successfully adapted for many metabolic reconstructions including human and yeast, might help researchers to understand the ciliate differences at genera, species, subspecies and populations level. Furthermore, with a combination of shared resources, it will be possible to study full ecosystems (food chain from endosymbionts, associated bacteria, predators-preys). These models, coupled with multi-omic approaches [40], could also highlight aspects of evolutionary biology and biogeography, sympatric and allopatric speciation.

## Methods

### Tetrahymena model (CiliateGEM) reconstruction

*CiliateGEM* was created using the protocol described by Thiele and Palsson [13]. First, AUGUSTUS (a gene prediction web server [41]), was used to identify all the *Tetrahymena* protein-encoding genes from the macronuclear genome assembly and then to assign the functional roles through accurate annotation with EC (Enzyme Commission) number. Next, a list of metabolic reactions was generated from KEGG and converted into a stoichiometric matrix. KEGG was used to search for Tetrahymena pathways and all the reactions were manually assembled. The specific links are listed in Additional file 3. A general list of exchange reactions was obtained from the literature [42]. The reaction list was reduced and adapted to fit *Tetrahymena* metabolism (Additional file 4). Establishing enzyme-reaction relationship is a complex step because an enzyme can be encoded by one or many genes, and each enzyme can catalyse one or more reactions [43]. We defined the growth media and external factors based on *Tetrahymena* experimental studies [28–30]. This facilitated us in capturing the organism physiological properties. The produced model was then converted into a mathematical one used to simulate the maximal growth with flux-balance analysis (FBA) [44].

FBA requires reactions to be represented as a stoichiometric matrix (S), with dimensions of m × n, where the metabolites (m) are represented as rows and the reactions (n) are represented as columns. The stoichiometric matrix is a numerical matrix of stoichiometric coefficients for each metabolite participating in a reaction. FBA assumes the system to be in a pseudo-steady state *S* ⋅ *v* = 0 holding for internal metabolites (reactants and products of the chemical reactions), where the vector *v* represents the flux distribution. An internal metabolite constitutes the model but cannot be imported or exported directly. An “exchange” counterpart of that metabolite, and an exchange reaction are required for modelling import/export reactions. Exchange metabolites can be imported and exported from the system, so they do not satisfy the steady state assumption. The flux distribution *v* is a vector of reaction rates and represents a feasible flux of metabolites through the reaction network, where under the principle of mass conservation, the total amount of metabolite consumed and total amount of metabolite produced are equal to zero.

In FBA, directionality and capacity constraints are placed on individual reactions by defining the upper (*Vmax*) and lower (*Vmin*) bounds on the range of values that the flux of each reaction can have (*Vmin* ≤ ***v*** ≤ *Vmax*). These constraints define the space of allowable flux distributions at which every metabolite is consumed or produced by each reaction in the system. Such flux bounds, coupled with gene-protein-reaction association rules, can also be used to map multiple environmental conditions, using binary threshold-based or continuous valve-based approaches [45, 46]. Despite these constraints, the system is still underdetermined (more unknowns than equations) and, as a result, infinite viable solutions exist. A flux distribution is obtained by defining an objective function that is a scalar product of the vector of flux rates *v*, and a vector of weights *c*, measuring how each component in the network contributes to the production of a biologically desirable phenotype.

Formally, we adopted the following linear program:

$$ \max\ c\cdotp v,\mathrm{such}\ \mathrm{that} $$

$$ S\cdot v=\dot{x} $$

$$ V\mathit{\min}\le v\le V\mathit{\max} $$

$$ {\dot{x}}_l=0\kern0.24em if\;{M}_i\in \mathrm{internal}\kern0.17em \mathrm{metabolites} $$

$$ {\dot{x}}_l\in R\kern0.24em if\kern0.28em {M}_i\kern0.28em \in \kern0.28em \mathrm{exchange}\kern0.34em \mathrm{metabolites} $$

*CiliateGEM*, consists of 545 reactions, 828 metabolites, 64 transport step reactions (which represent the import and export of metabolites between extra- and intra-cellular space), and 84 boundary steps (which represent the input and output of metabolites from extracellular space and a biomass reaction). The biomass reaction defines a unique objective function to effective growth. This describes the rate at which all of the biomass precursors are made in the correct proportions. Linear programming was used to calculate the optimal flux distribution that maximizes growth. The model was encoded in SBML format [47] and fulfils MIRIAM requirements [48]. It was then imported in MATLAB (version R2016b). The simulation was carried out using COBRA [49] toolbox with the linear programming solver GLPK. All biomass flux values were given in millimoles/hour/grams of dry weight (mmol/gDW/hr). CiliateGEM is provided as Additional files (Additional file 5: CiliateModel.mat).

## Additional files

Additional file 1: Databases and bioinformatics resources for ciliates [50–54]. (DOCX 14 kb)

Additional file 2: List of organelles found in most ciliates. (DOCX 13 kb)

Additional file 3: Links of the KEGG reactions. (XLSX 11 kb)

Additional file 4: List of reactions. (XLSX 98 kb)

Additional file 5: Simulation file. (XLSX 226 kb)

Additional file 6: SBML version of the model. (XML 716 kb)

Additional file 7: Matlab version of the model. (MAT 48 kb)

## Abbreviations

COBRA: The COnstraint-Based Reconstruction and Analysis Toolbox
FBA: Flux Balance Analysis
GEM: Genome-scale models
MMI: Marine Microbiology Initiative
