# Supplementary material for: CiliateGEM: an open-project and a tool for predictions of ciliate metabolic variations and experimental condition design
Source: BMC Bioinformatics. 2018 Nov 30;19(Suppl 15):442. doi: 10.1186/s12859-018-2422-9 (PMC6266953; doi:10.1186/s12859-018-2422-9)
Supplement: Supplementary file 1 — Databases and bioinformatics resources for ciliates [50–54]. (DOCX 14 kb) [file 12859_2018_2422_MOESM1_ESM.docx]

**Additional file 1: Databases and bioinformatics resources for ciliates (all accessed 8 Aug 2018).**

1- *Tetrahymena* Genome Database Wiki (TGD [50]: <http://ciliate.org/index.php/home/welcome>) is a user-updatable database providing macronuclear genomic sequences for *T. thermophila* (and additional *Tetrahymena* species) determined at the Institute for Genomic Research (TIGR). The web site provide bioinformatics resources <http://ciliate.org/index.php/downloads/cgc/files/BIOINFORMATICS%20MODULE> for genome analysis. It also reports all the news on *Tetrahymena* genome, genes, and proteins collected from scientific literature and the research community.

*2- Tetrahymena* functional genomics database (TetraFGD: <http://ciliate.org/index.php/downloads/cgc/files/BIOINFORMATICS%20MODULE>) provides user-friendly search functions that assist researchers in accessing gene models, transcripts, gene expression data and gene–gene relationships. The database contains four major resources: the rna-seq transcriptome, microarray analysis, gene networks and phosphoproteome. The microarray analysis describes the gene expression during three major stages of the *T. thermophila* life cycle, i.e. growth, starvation and conjugation; the gene network data identify potential gene–gene interactions of 15 049 genes. The phosphoproteome data were obtained by mass spectrometry (MS)-based analysis and cover the three different physiological/developmental stages in *Tetrahymena thermophila* life cycle, as reported above.

*3*-ParameciumDB (<http://paramecium.cgm.cnrs-gif.fr/>): a database associated with the genome sequencing project of the model ciliate Paramecium. It contains the macronuclear genome sequence and annotations, linked to available genetic data (RNA interference data and stock collection). The web site provides tools that include BioMart for complex queries, GBrowse2 for genome browsing, the Apollo genome editor for expert curation of gene models. Furthermore, a Blast server, a motif finder and tools for the evaluation of off-target RNAi matches are also available [51, 52].

4- OxyDB (/<http://oxy.ciliate.org/index.php/home/welcome>): it provides information on the genome, genes, and proteins of *Oxytricha trifallax* [53]. The web site also provides bioinformatics resources for genome analysis.

5- <mds_ies_db>: (http://oxytricha.princeton.edu/mds_ies_db) is a database of genome recombination and rearrangement annotations, and it provides tools for visualization and comparative analysis of precursor and product genomes. The database contains annotations for *O. trifallax* and *T. thermophila* [54].

6- Marine Microbial Eukaryote Transcriptome Sequencing Project: a database containing 750 transcriptomes from some of the more abundant and ecologically significant microbial eukaryotes in the ocean, including ciliates [22].

7- The genome from the Antarctic ciliate *Euplotes focardii* is available at

<https://www.ncbi.nlm.nih.gov/genome/50465?genome_assembly_id=293471>

8- *Euplotes crassus* proteome (<https://github.com/vladpetyuk/EuplotesCrassus.proteome>).
