# Supplementary material for: CiliateGEM: an open-project and a tool for predictions of ciliate metabolic variations and experimental condition design
Source: BMC Bioinformatics. 2018 Nov 30;19(Suppl 15):442. doi: 10.1186/s12859-018-2422-9 (PMC6266953; doi:10.1186/s12859-018-2422-9)
Supplement: Supplementary file 2 — List of organelles found in most ciliates. (DOCX 13 kb) [file 12859_2018_2422_MOESM2_ESM.docx]

**Additional file 2.** **List of organelles found in most ciliates.** Beside the compartments typical of all the eukaryotic cells (different compartments inside the mitochondria, Golgi apparatus, Endoplasmic reticulum, etc) ciliates have characteristic organelles.

| **Organelles** | **Function** |
| --- | --- |
| **Micronucleus (MIC)** | Specialized for sexual exchange during conjugation. No transcriptionally active. No nucleoli. |
| **Macronucleus (MAC)** | It derives from the MIC after extensive rearrangement during conjugation. Specialized for transcription. It possesses nucleoli. |
| **Oral apparatus** | Cortical feeding structure composed of ciliated and non-ciliated basal bodies interconnected by a framework microtubules and filaments used for catching food from the environments. |
| **Food vacuole** | Membrane-bound sacs responsible for phagocytosis. They form from the oral apparatus to introduce food particle inside the cell. Digestion occurs after fusing with lysosomes. |
| **Contractile vacuole** | Involved in osmoregulation |
| **Anal pore** | Responsible for waste excretion after food digestion |
| **Cilia** | Specialized microtubule appendices used for locomotion in the whole body or for feeding in the cytostome. |
| **Infraciliature** | A part of the cytoskeleton that provides a scaffolding for cilia and determines the form of the body. |
| **Spasmonemes or myonemes** | Contractile organelles energized by calcium ion binding, not by ATP hydrolysis. |
| **Extrusomes** | Ejectable membrane-bound organelles. |
